# UCP3 Regulates Single-Channel Activity of the Cardiac mCa1

**DOI:** 10.1007/s00232-016-9913-2

**Published:** 2016-07-01

**Authors:** Lukas J. Motloch, Tina Gebing, Sara Reda, Astrid Schwaiger, Martin Wolny, Uta C. Hoppe

**Affiliations:** Department of Internal Medicine II, Paracelsus Medical University, Muellner Hauptstr. 48, A-5020 Salzburg, Austria

**Keywords:** UCP3, Mitochondria, Calcium, Mitochondrial calcium uniporter, mCa1, ATP

## Abstract

Mitochondrial Ca^2+^ uptake (mCa^2+^ uptake) is thought to be mediated by the mitochondrial Ca^2+^ uniporter (MCU). UCP2 and UCP3 belong to a superfamily of mitochondrial ion transporters. Both proteins are expressed in the inner mitochondrial membrane of the heart. Recently, UCP2 was reported to modulate the function of the cardiac MCU related channel mCa1. However, the possible role of UCP3 in modulating cardiac mCa^2+^ uptake via the MCU remains inconclusive. To understand the role of UCP3, we analyzed cardiac mCa1 single-channel activity in mitoplast-attached single-channel recordings from isolated murine cardiac mitoplasts, from adult wild-type controls (WT), and from UCP3 knockout mice (UCP3^–/–^). Single-channel registrations in UCP3^−/−^ confirmed a murine voltage-gated Ca^2+^ channel, i.e., mCa1, which was inhibited by Ru360. Compared to WT, mCa1 in UCP3^−/−^ revealed similar single-channel characteristics. However, in UCP3^−/−^ the channel exhibited decreased single-channel activity, which was insensitive to adenosine triphosphate (ATP) inhibition. Our results suggest that beyond UCP2, UCP3 also exhibits regulatory effects on cardiac mCa1/MCU function. Furthermore, we speculate that UCP3 might modulate previously described inhibitory effects of ATP on mCa1/MCU activity as well.

## Introduction

Mitochondrial Ca^2+^ handling is involved in several major cellular processes. It is known to regulate the rate of mitochondrial energy adenosine triphosphate (ATP) production (Jouaville et al. 1999; Territo et al. 2000), to control mitochondrial reactive oxygen species (ROS) generation (Kohlhaas et al.), and to supervise the initiation of cell death (Bernardi and Rasola 2007; Hoppe; O’Rourke 2007). Furthermore, it is supposed to modulate the profile of intracellular Ca^2+^ signaling (Kirichok et al. 2004; O’Rourke, 2007; Rizzuto et al. 1998).

Mitochondrial Ca^2+^ uptake (mCa^2+^ uptake) is mainly mediated by the mitochondrial calcium uniporter (MCU) (Kirichok et al. 2004; Maack et al. 2006; Michels et al. 2009) which is highly sensitive to Ruthenium Red or to its more specific derivate Ruthenium360 (Ru360) (Brookes et al. 2008; Kirichok et al. 2004; Michels et al. 2009; Zazueta et al. 1999). The MCU was identified as a highly Ca^2+^-selective protein complex that consists of the pore-forming mitochondrial Ca^2+^ uniporter protein (MCU) (Baughman et al. 2011; Chaudhuri et al. 2013; De Stefani et al. 2011), the essential MCU regulator (EMRE), and the mitochondrial calcium uptake 1 and 2 (MICU1/2) (Ahuja and Muallem 2014; Mallilankaraman et al. 2012; Perocchi et al. 2010; Plovanich et al. 2013). However, the function and regulatory mechanisms of MCU have not been fully understood yet.

In recent studies performed in mitoplasts isolated from HeLa cells, three mitochondrial Ca^2+^ channels have been identified (Bondarenko et al. 2013, 2014). Among them, the *i*-MCC was proven to display MCU- and MCU/EMRE-dependent activity, indicating it as an MCU-established current (Bondarenko et al. 2014). The single-channel characteristics of the channel correspond to the ruthenium sensitive cardiac current mCa1, which is supposed to underlie the MCU in the human and the murine heart (Bondarenko et al. 2013, 2014; Michels et al. 2009; Motloch et al. 2016).

Uncoupling proteins (UCPs) are found in the inner mitochondrial membrane and belong to a superfamily of mitochondrial ion transporters (Arsenijevic et al. 2000). UCP2 and UCP3 are expressed in cardiac tissue (Alan et al. 2009). In the heart, these isoforms regulate cardiac ROS production and therefore reduce ischemia reperfusion injury (Chen et al. 2015; McLeod et al. 2005; Ozcan et al. 2013; Perrino et al. 2013; Safari et al. 2014; Teshima et al. 2003; Toime and Brand 2010). Recently, UCP2 was also shown to modulate cardioprotective effects of MCU inhibition (Motloch et al. 2015). However, the impact of both proteins on mCa^2+^ uptake remains still a matter of debate (Brookes et al. 2008; Trenker et al. 2007). In recent years, several studies have provided further evidence that indeed UCP2 and UCP3 are able to regulate mitochondrial Ca^2+^ handling (Waldeck-Weiermair et al. 2010a, b, 2011, 2013). In isolated mitoplasts, UCP2 was proven to modulate characteristics of mitochondrial Ca^2+^ channels (Bondarenko et al. 2015; Motloch et al. 2016) including the cardiac mCa1 (Motloch et al. 2016). Furthermore, in the heart, the protein was observed to mediate inhibitory effects on mCa1/MCU by ATP and to provoke changes of excitation–contraction coupling (Motloch et al. 2016).

However, if UCP3 may also modulate mCa1/MCU activity in the heart remains inconclusive. Therefore, to clarify this issue, we evaluated electrophysiological properties of the mCa1 in cardiac mitoplasts from UCP3^–/−^ mice and wild-type controls (WT). Our data reveal that similar to findings obtained in UCP2^−/−^ mice, UCP3 modulates the channel’s activity in an ATP-dependent manner.

## Materials and Methods

### Animals

Animals were euthanized by cervical dislocation. For control experiments WT hearts were obtained from 12- to 16-week-old male C57BL/6 mice which were purchased from Charles River Laboratories, Research Models and Services, Germany. Male and female *Ucp3* knockout mice (UCP3^−/−^) (Gong et al. 2000) were crossed 10 times into the C57BL/6 background. *Ucp3* ablation was confirmed in tails by PCR analysis of the genomic *Ucp3* (Toime and Brand 2010). Male animals were sacrificed at age 12–16 weeks.

Animals were housed in the facilities of the Paracelsus Medical University, Salzburg. The implementation of the experiments conformed to the Guide for the Care and Use of Laboratory Animals published by the US National Institutes of Health (NIH publication No. 85-23, revised 1996).

### Preparation of Mitoplasts

Isolated intact cardiac subsarcolemmal mitoplasts were prepared from isolated myocytes by differential centrifugation, as previously reported (Er et al. 2004; Kirichok et al. 2004; Michels et al. 2009; Motloch et al. 2016). Briefly, hearts were obtained from WT or UCP3^−/−^ mice and single ventricular myocytes were isolated from murine hearts by enzymatic digestion, as previously described (Hoppe and Beuckelmann 1998; Lange et al. 2003; Michels et al. 2009; Motloch et al. 2016). Freshly isolated cardiomyocytes were used within 1–2 h. Myocytes were labeled with Mitotracker Green 1 μM (Life Technologies, Carlsbad, CA, USA) to facilitate identification of intact mitoplasts after further subcellular purification (Er et al. 2004; Kirichok et al. 2004; Michels et al. 2009; Motloch et al. 2016). Mitochondria were stored at 4 °C for up to 24 h for patch-clamp experiments. Mitoplasts were prepared from intact mitochondria prior to patching or protein preparation by osmotic shock, as previously described (Er et al. 2004; Kirichok et al. 2004; Michels et al. 2009; Motloch et al. 2016).

### Single-Channel Recordings

All experiments were performed in the mitoplast-attached configuration of the patch-clamp technique (at least 60 test pulses of 150-ms duration at 1.67 Hz, if not indicated otherwise; sampling frequency, 10 kHz; corner frequency, 2 kHz), as previously reported (Michels et al. 2009; Motloch et al. 2016). The bath solution contained 160 mM KCl, 10 mM HEPES, 1 mM EDTA, 1 mM EGTA, pH 7.2 adjusted with KOH. Pipettes were filled with a solution containing 105 mM CaCl_2_, 10 mM HEPES, pH 7.2 adjusted with Ca(OH)_2_ (Er et al. 2004; Kirichok et al. 2004; Michels et al. 2009; Motloch et al. 2016). Specific drugs were added to the solutions to block the mitochondrial permeability transition pore (10 µM cyclosporine A, Sigma Aldrich, St. Louis, MO, USA), the mitochondrial ryanodine receptor (10 µM dantrolene, Sigma Aldrich, St. Louis, MO, USA), the inositol triphosphate receptor (10 µM xestospongin C, Sigma Aldrich, St. Louis, MO, USA), and the mitochondrial Na^+^–Ca^2+^ exchanger (10 µM CGP-37157, Calbiochem, San Diego, CA, USA) (Er et al. 2004; Kirichok et al. 2004; Michels et al. 2009; Motloch et al. 2016). Ru360 (10 µM, Merck, Darmstadt, Germany) and ATP (1 mM, Sigma Aldrich, St. Louis, MO, USA) were added to the bath solution as indicated. Currents were recorded and digitized with an Axopatch 200B amplifier and Digidata 1200 interface (MDS Analytical Technologies, Toronto, Canada), as previously described (Gassanov et al. 2006; Lange et al. 2003; Michels et al. 2009; Motloch et al. 2016).

### Single-Channel Analysis

Single-channel analysis was done using custom software only from one-channel patches, as previously reported (Er et al. 2004; Michels et al. 2009; Motloch et al. 2016). Briefly, linear leak and capacity currents were digitally subtracted using the average currents of nonactive sweeps. For detailed gating analysis, idealized currents were analyzed in 150 ms steps. Active sweeps were defined as those with at least one opening. The total open probability (Po, total; defined as the occupancy of the open state during the total pulse duration) was analyzed for at least 3 s pulse durations at −100 mV of 60 sweeps with 150 ms duration. Single-channel amplitudes were determined by direct measurements of fully resolved openings for conductance calculations or as the maximum of Gaussian fits to amplitude histograms (Michels et al. 2009; Motloch et al. 2016).

### Statistical Analysis

N refers to the number of patch-experiments obtained in mitoplasts isolated from a minimum of three hearts. Pooled data are presented as mean ± SEM. Comparisons between groups were performed with one-way ANOVA followed by post hoc Tukey test. Probability values of *P* < 0.05 were regarded significant.

## Results

### Characterization of mCa1 Single-Channel Activity in Subsarcolemmal, Cardiac Mitoplasts from UCP3^−/−^

**Fig.1:**
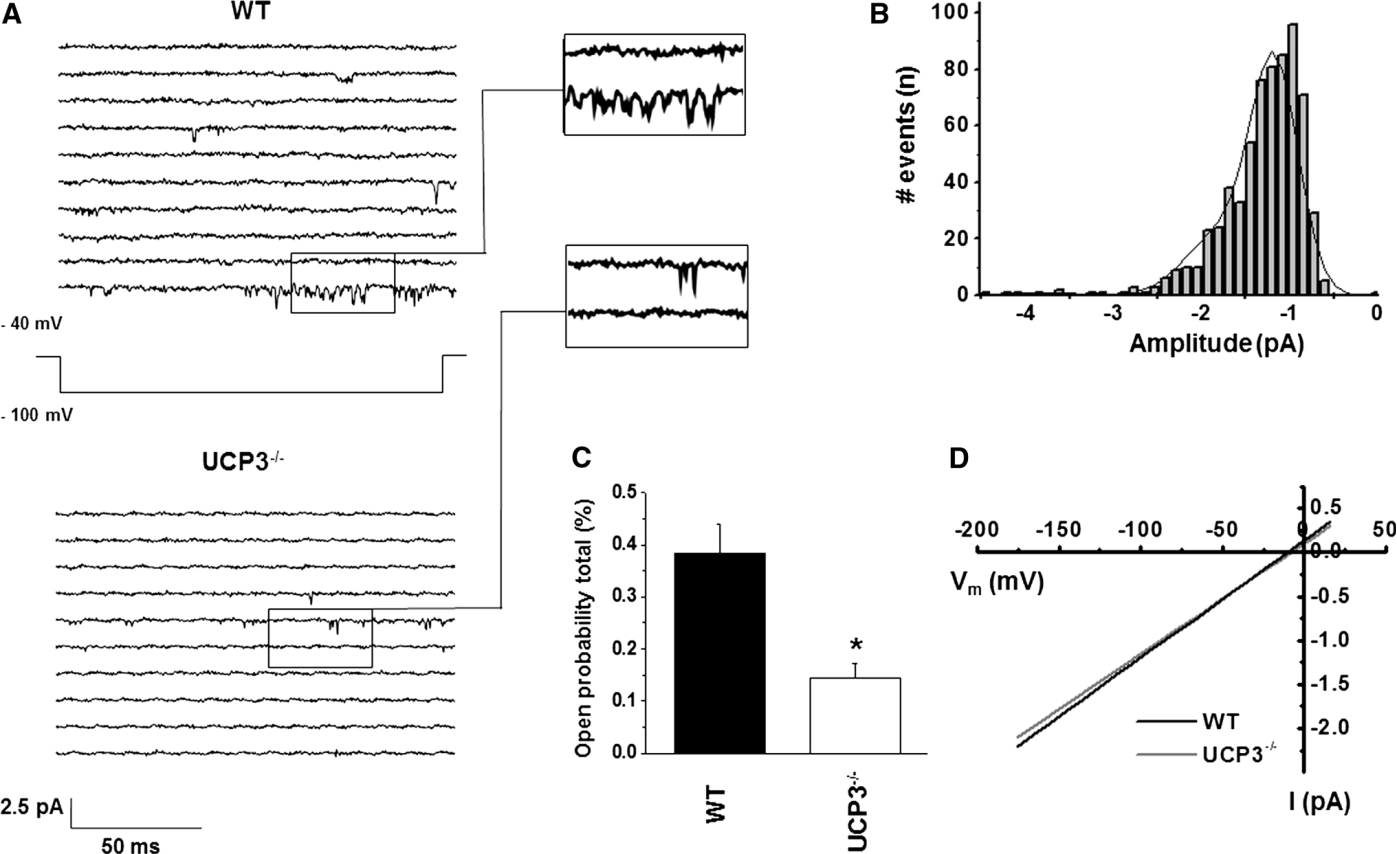
**a** Examples of consecutive original traces of cardiac mCa1 in mitoplasts from WT and UCP3^−/−^. **b** Amplitude histogram of mCa1 in mitoplasts from UCP3^−/−^. mCa1 in UCP3^−/−^ showed three amplitude levels with −1.16 pA being the most common observed amplitude [I_unitary_: −1.16 ± 0.05 pA, *n* = 21; µ_1_:−1.17 pA (71 %), µ_2_: −1.80 pA (28 %), µ_3_: −3.72 pA (1 %)]. **c** Total open probability (Po, total) of mCa1 in WT and UCP3^−/−^: In comparison to WT, mCa1, Po, total of mCa1 in UCP3^−/−^ were significantly decreased (*P < 0.05). **d** Slope conductance of mCa1 in WT (13.10 ± 1.42 pS, *n* = 10) and of mCa1 in UCP3^−/−^ (12.55 ± 1.22 pS, *n* = 14) was not different

To clarify the role of UCP3 in cardiac mCa^2+^ uptake we analyzed single-channel currents in cardiac mitoplasts from UCP3^−/−^ mice. By patch-clamping the inner membrane of subsarcolemmal mitoplasts prepared from isolated cardiomyocytes from UCP3^−/−^, we verified the existence of murine mitochondrial mCa1 channels in 29 % of total patches. Compared to WT (21 %) UCP3^−/−^ showed a trend towards an increase in the probability of occurrence (ratio of active to total patches), however, without reaching statistical significance (*P* = 0.20, calculated by Chi Square test). In UCP3^−/−^ we detected voltage-dependent single-channel currents with a unitary conductance of 12.55 ± 1.22 pS, three different amplitude sublevels with −1.16 ± 0.02 pA being the most common, and total open probability of 0.14 ± 0.02 % (Po, total at −100 mV; Fig. 1; Table 1). Notably, neither channel’s conductance nor the amplitude was different from WT (Table 1; Fig. 1d). However, compared to WT, UCP3^−/−^ presented a significant reduction in total open probability and in mean open time, indicating mCa1 to be present but less active in UCP3^−/−^ cardiac mitochondria (Fig. 1; Table 1).

**Table 1:** Gating parameters of mCa1 in WT and UCP3^−/−^

	WT	UCP3^−^/^−^	UCP3^−^/^−^ + Ru360 (10 µM)	UCP3^−^/^−^ + ATP (1 mM)
Total open probability (%)	0.38 ± 0.04	0.14 ± 0.02*	0.04 ± 0.01*^#^	0.10 ± 0.03*
Mean open time (ms)	0.36 ± 0.02	0.25 ± 0.01*	0.24 ± 0.05*	0.27 ± 0.01
Mean close time (ms)	7.11 ± 0.87	10.64 ± 0.75	15.72 ± 4.32*	15.66 ± 5.07*
Mean first latency (ms)	56.73 ± 2.32	59.52 ± 2.60	66.30 ± 6.36	58.81 ± 5.25
Amplitude/I_unitary_ (pA)	−1.20 ± 0.03	−1.16 ± 0.02	−1.14 ± 0.03	−1.16 ± 0.03
No. Experiments	15	21	5	4

Gating parameters of mCa1 in cardiac WT and UCP3^−/−^ mitoplasts as well as in the presence of Ruthenium 360 (Ru360; 10 µM) or ATP (1 mM) in UCP3^−/−^. Holding potential −40 mV, test potential −100 mV

*P < 0.05 vs. WT; ^#^ *P* < 0.05 vs. UCP3^−/−^ control

### Characterization mCa1 Single-Channel Activity in Subsarcolemmal Cardiac Mitoplasts from UCP3^−/−^ ± Ru360 and ATP

**Fig.2:**
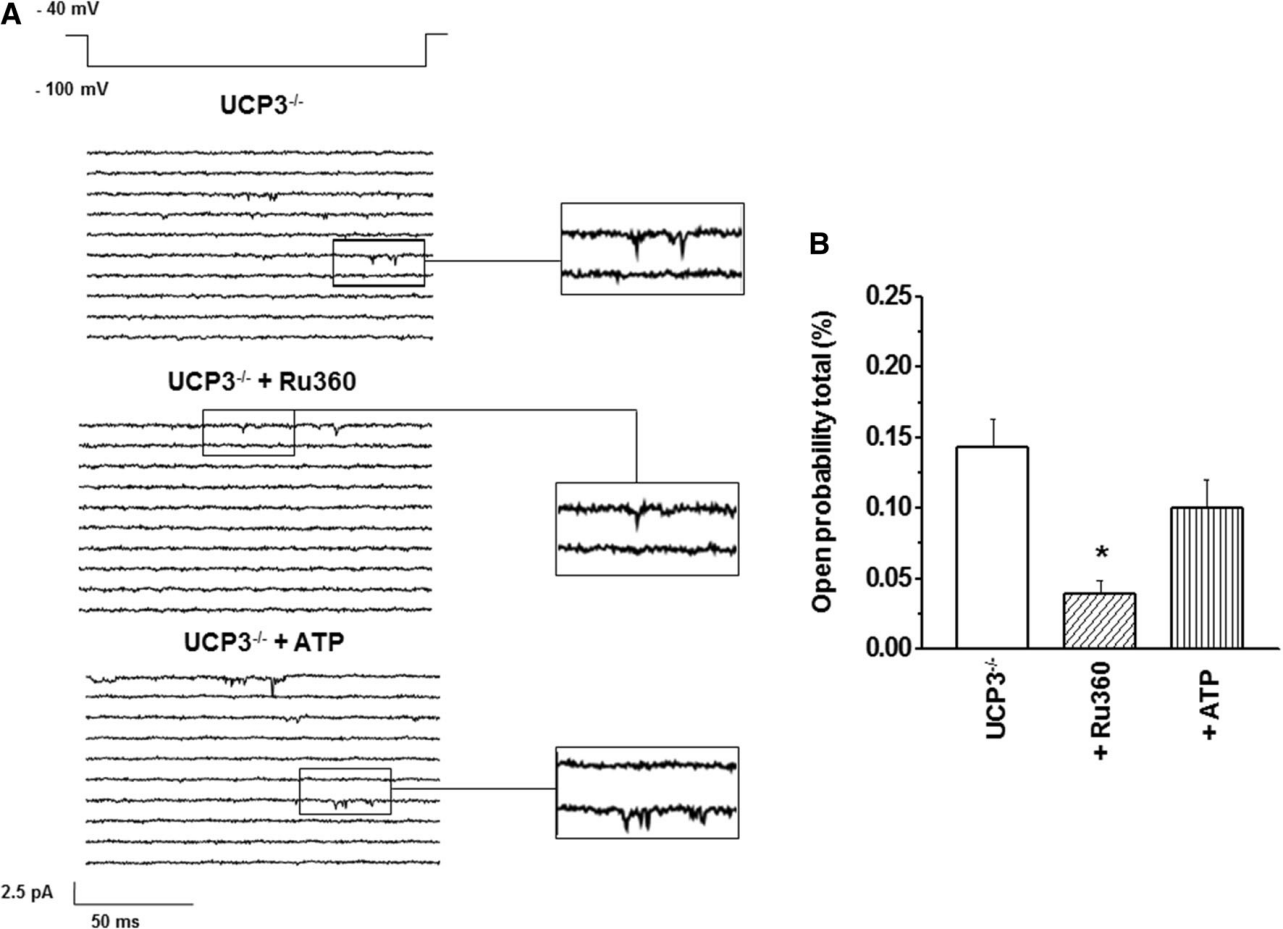
**a** Examples of consecutive original traces of cardiac mCa1 in mitoplast from UCP3^−/−^: mCa1 in UCP3^−/−^ control versus mCa1 in UCP3^−/−^ + Ru360 versus mCa1 in UCP3^−/−^ + ATP. **b** In cardiac mitoplasts from UCP3^−/−^ Ru360 (10 µM, *n* = 5) significantly decreased total open probability (Po, total) of mCa1 (*P < 0.05). ATP (1 mM, *n* = 4) had no significant effect on mCa1 activity in UCP3^−/−^

mCa1/MCU activity is known to be inhibited by Ru360 (Kirichok et al. 2004; Michels et al. 2009; Motloch et al. 2016). Therefore, mCa1 sensitivity to Ru360 in UCP3 was evaluated (Fig. 2; Table 1). Consistent with data recently obtained in murine cardiac WT mitoplasts (Motloch et al. 2016), Ru360 significantly decreased the total open probability (Po, total: 0.04 ± 0.01 %, *n* = 5, *P* < 0.05) without affecting further gating characteristics of the channel (Fig. 2; Table 1).

Since ATP was reported to inhibit cardiac mCa1 activity in a UCP2-dependent manner (Motloch et al. 2016), we speculated similar effects for UCP3. Thus, we performed additional experiments using ATP in UCP3^−/−^. After application of ATP, no significant changes in total open probability were observed (Po, total = 0.10 ± 0.03 %, *n* = 4, *P* = 0.55, Fig. 2; Table 1). In addition, no further single-channel characteristics were influenced by the nucleotide (Fig. 2; Table 1).

## Discussion

The possible role of UCP2 and UCP3 in modulating mCa^2+^ uptake is a matter of debate (Brookes et al. 2008; Trenker et al. 2007). In a recent study, UCP2 was shown to regulate cardiac mCa^2+^ uptake by modulating mCa1/MCU single-channel activity (Motloch et al. 2016). However, the role of UCP3 in regulating cardiac mitochondrial Ca^2+^ handling remains still inconclusive. Therefore, to define the role of UCP3, we performed single-channel recordings of subsarcolemmal mitoplasts prepared from isolated cardiomyocytes in WT and UCP3^−/−^. In UCP3^−/−^ mitoplasts we detected a murine cardiac Ca^2+^ channel which presented burst like open states, a mean amplitude of −1.1–1.2 pA at −100 mV, and a conductance of 12–13 pS. These gating characteristics are specific for the murine and human mCa1 which correspond to the *i*-MCC as previously described in HeLa cells (Bondarenko et al. 2013, 2014; Michels et al. 2009; Motloch et al. 2016). Of note, the channel’s activity characteristics have been described to be indicative for the MCU-established current (Bondarenko et al. 2013, 2014; Michels et al. 2009; Motloch et al. 2016). Consistent with these observations, in our study mCa1 currents in UCP3^−/−^ were shown to be sensitive to Ru360 (Fig. 2; Table 1), which is known to block specifically the MCU (Kirichok et al. 2004; Zazueta et al. 1999). Therefore, despite earlier suggestions (Trenker et al. 2007), our results indicate, that UCP3 may not be essential for the MCU-established mitochondrial Ca^2+^ uptake. This conclusion is consistent with recent reports. The MCU was characterized as a highly Ca^2+^-selective protein complex that consists of the pore-forming mitochondrial Ca^2+^ uniporter protein (MCU) (Baughman et al. 2011; Chaudhuri et al. 2013; De Stefani et al. 2011), the essential MCU regulator (EMRE), and further regulatory proteins the mitochondrial calcium uptake 1 and 2 (MICU1/2) (Ahuja and Muallem 2014; Mallilankaraman et al. 2012; Perocchi et al. 2010; Plovanich et al. 2013).

However, in our study compared to WT, we detected a decreased single-channel activity in UCP3^−/−^ mitoplasts. A decrease in mCa^2+^ uptake after UCP3 depletion is consistent with previous reports obtained in intact and permeabilized HeLa as well as endothelial cells (Ahuja and Muallem 2014; Trenker et al. 2007; Waldeck-Weiermair et al. 2010a, b, 2011, 2013). Our data extend these observations to the heart and furthermore reveal a physiological mechanism. We suggest that in the heart and probably also in other tissues, the protein influences mCa^2+^ uptake by modulating mCa1/MCU single-channel activity. Therefore, our results might implicate that, as previously speculated for UCP2 (Motloch et al. 2016), UCP3 could operate cooperatively or sequentially for the modulation of transporting Ca^2+^ across the inner mitochondrial membrane as another part of the cardiac MCU complex system.

However, regulatory effects on further mCa^2+^ uptake mechanisms should be considered too. Of note, in respect to UCP2 function, a recent study in HeLa cells detected the protein to regulate the extra-large mitochondria calcium channel (*xl*-MCC). This channel essentially contains MCU and EMRE. Notably, MCU was shown to have a much higher affinity to *xl*-MCC than to other MCU-dependent calcium currents including *i*-MCC (Bondarenko et al. 2015). An alternative explanation as how UCP3 might regulate mCa^2+^ uptake was reported by De Marchi and colleagues. The authors observed a modulation of mitochondrial Ca^2+^ by increased ATP and elevated SERCA activity leading to reduced depletion of the internal Ca^2+^ stores in intact HeLa cells under UCP3 depletion (De Marchi et al. 2011).

Altered UCP3 expression was demonstrated to influence cytosolic Ca^2+^ handling. In endothelial cells, distinct sites in the intermembrane loop 2 of the UCP3 protein were reported to modulate cytosolic and mitochondrial Ca^2+^ handling by sequestration of Ca^2+^ preferably released from the endoplasmatic reticulum (Waldeck-Weiermair et al. 2010a, b). In respect to cardiac tissue, changes in mCa^2+^ uptake in UCP2 knock down mice were shown to influence cytosolic Ca^2+^ handling by presenting a diminished trasnsarcolemmal Ca^2+^ influx in these animals (Motloch et al. 2016). Therefore, one might speculate that in the heart cytosolic Ca^2+^ handling might also be regulated by UCP3. However, these suggestions were not the matter of investigation in the present study and need to be addressed in further trials.

Previous studies also described inhibitory effects of adenine nucleotides especially of ATP on mCa^2+^ uptake (Litsky and Pfeiffer 1997) and on UCP activity, respectively (Echtay et al. 2001; Zackova et al. 2003). Indeed, recently ATP was shown to influence single-channel characteristics of mCa1 by reducing its total open probability without affecting further gating parameters (total open probability: WT 0.34 ± 0.05 vs. WT+ATP 0.06 + 0.01; *P* < 0.05; detailed gating characteristics are presented in Table 1 in Motloch et al. 2016). However, no effects were observed in UCP2^−/−^ murine cardiac mitoplasts (total open probability: UCP2^−/−^ 0.08 ± 0.02 vs. UCP2^−/−^+ATP 0.06 + 0.01; *P* > 0.05; detailed gating characteristics are presented in Table 2 in Motloch et al. 2016). These observations were reassured in isolated cardiomyocytes indicating UCP2 to influence inhibitory effects of ATP on mCa1/MCU activity (Motloch et al. 2016). Therefore, we further decided to explore the impact of ATP in UCP3^−/−^. In this study, with a number of four experiments, no significant effects of ATP on mCa1 activity were observed in UCP3^−/−^ mice (total open probability: UCP3^−/−^ 0.14 ± 0.02 vs. UCP3^−/−^+ATP 0.10 + 0.03; *P* > 0.05; Table 1). This observation might support the notion that the inhibition of mCa^2+^ uptake by ATP might be mediated also via UCP3. However, regarding the limited number of experiments obtained by a challenging research technique in isolated cell organelles in this study, these conclusions should be applied with caution. Further studies, should confirm these speculations on the entire cell level.

Nevertheless, our results might be supported by a previous mentioned report from De Marchi and colleagues. Using histamine and boosting intracellular Ca^2+^ concentrations, the authors noted an increase in mitochondrial ATP concentrations in UCP3-depleted HeLa cells (De Marchi et al. 2011). They speculated, that a decline in uncoupling mechanisms and therefore a more efficient ATP synthase function might be responsible for their observations (De Marchi et al. 2011). Our data might provide an alternative explanation. MCU function was observed to be dependent on intracellular ATP concentrations (Motloch et al. 2016). In addition, the MCU was reported to be involved in the regulation of mitochondrial energy ATP production (Jouaville et al. 1999; Territo et al. 2000). Thus, one might speculate that under physiological conditions UCP3 (and probably also UCP2) seems to serve as a regulator of the respiratory chain via the modulation of mCa^2+^ uptake indicating a physiological feedback mechanism in situations with high energy demands. However, in contrast to our data as well as in contrast to previous results obtained in permeabilized HeLa and endothelial cells (Trenker et al. 2007; Waldeck-Weiermair et al. 2010a, 2011) De Marchi and colleagues failed to record any differences in mCa^2+^ uptake in UCP3-depleted permeabilized cells (De Marchi et al. 2011). A possible explanation for their findings might be the usage of higher Ca^2+^ concentrations which could also activate further mCa^2+^ uptake mechanisms (Brustovetsky and Klingenberg 1996; Hoppe 2010; O’Rourke 2007).

How UCP3 and also as previously described UCP2 (Motloch et al. 2016) might potentially prevent the inhibitory effect of ATP and regulate mCa1 channel gating are not fully understood yet. Nevertheless, previous findings might contribute to some speculations. Both mitochondrial proteins are postulated to have a nucleotide-binding site which promotes inhibitory effects of ATP (Ricquier and Bouillaud 2000; Zackova et al. 2003). Therefore, ATP is described to decreased UCP-mediated proton leak. This mechanism could possibly promote local increases in proton concentration in the intermembrane space. Assuming that MCU and UCP2 or UCP3 might be colocalized in the inter mitochondrial membrane, one might speculate that the local change in proton concentrations would be able to influence the channel’s activity. Of note, MCU function is known to be pH dependent (Reed and Bygrave 1975). However, further mechanisms should also be considered. By studying chimera constructs, Waldeck-Weiermair and colleagues elegantly demonstrated that distinct sites in the intermembrane loop 2 of UCP3 affect mitochondrial uptake of high and low calcium signals (Waldeck-Weiermair et al. 2010a). Of note, these specific sequences are not found in UCP1 (Waldeck-Weiermair et al. 2010a). Their observations support the idea of a more complex regulatory mechanism. Therefore, one might further assume that specific sites of UCP2 or UCP3 could directly interact with or as a part of the MCU complex to differentially influence the open state of the channel in an ATP-dependent manner. Nevertheless, these speculations were not the matter of the current investigations and need be addressed in further trials.

## Conclusion

In summary our study supports an essential role for UCP3 in modulating cardiac mCa^2+^ uptake via regulation of mCa1 single-channel activity. Furthermore, our data suggest that UCP3 might also modulate inhibitory effects of ATP on mCa1/MCU function.
